# Differential induction of activation and apoptosis by TCR signaling in sooty mangabeys and rhesus macaques

**DOI:** 10.1186/1742-4690-9-S2-P36

**Published:** 2012-09-13

**Authors:** S Yu, K Rogers, F Villinger, A Kaur

**Affiliations:** 1 Harvard Medical School, Southborough, USA; 2 Emory University, Atlanta, GA, USA

## Background

We previously showed that an increase in CD4+ T lymphocyte apoptosis and elevation of plasma tumor necrosis-receptor associated apoptosis-inducing ligand (TRAIL) occurs in rhesus macaques (RM) but not sooty mangabeys (SM) during acute SIV infection.

## Methods

To further examine the mechanisms underlying differential apoptosis in SIV-infected RM and SM, we compared the in vitro responses to TCR signaling in seven SIV-negative SM and seven SIV-negative RM. PBMC were cultured for 18 hours with cross-linked CD3 and CD28 and subsequently analyzed by flow cytometry for upregulation of CD69, TRAIL and active Caspase 3, and for production of cytokines.

## Results

Following TCR stimulation, there was a significant increase in TRAIL on T cell subsets, NK cells and myeloid DC cells (mDCs) in both species. However, levels of membrane TRAIL were significantly higher in CD8+ T lymphocytes and NK cells of SM compared to RM suggesting that they may be more cytotoxic on activation. TCR stimulation also resulted in an upregulation of CD69 and production of IFNγ, IL-2 and TNFα by T cell subsets in both species with greater levels being observed in SM. In contrast to SM, RM showed significantly higher frequencies of apoptotic mDCs both ex vivo and following TCR stimulation. In vivo inoculation of RM with SIVmac239 resulted in increased frequency of ex vivo apoptotic mDCs at 2-3 weeks post-infection. Increased apoptosis was also observed after overnight culture in medium but was abrogated by addition of soluble death receptor 5 indicating that it was TRAIL-mediated.

## Conclusion

Overall, these data show an increased susceptibility to apoptosis of mDCs in RM, and a disconnect between T cell activation and apoptosis in SM. Elucidating the mechanisms by which SM are protected from apoptosis will be important for understanding the basis of nonpathogenicity in natural hosts of SIV infection.

